# Effect of metformin on residual cells after chemotherapy in a human lung adenocarcinoma cell line

**DOI:** 10.3892/ijo.2013.2120

**Published:** 2013-10-03

**Authors:** SATORU KITAZONO, YUICHI TAKIGUCHI, HIRONORI ASHINUMA, MIYAKO SAITO-KITAZONO, ATSUSHI KITAMURA, TETSUHIRO CHIBA, EMIKO SAKAIDA, IKUO SEKINE, YUJI TADA, KATSUSHI KUROSU, SEIICHIRO SAKAO, NOBUHIRO TANABE, ATSUSHI IWAMA, OSAMU YOKOSUKA, KOICHIRO TATSUMI

**Affiliations:** 1 Departments of Respirology, Graduate School of Medicine, Chiba University, Chuo-ku, Chiba 260-8670, Japan; 2 Medical Oncology, Graduate School of Medicine, Chiba University, Chuo-ku, Chiba 260-8670, Japan; 3 Medicine and Clinical Oncology, Graduate School of Medicine, Chiba University, Chuo-ku, Chiba 260-8670, Japan; 4 Cellular and Molecular Medicine, Graduate School of Medicine, Chiba University, Chuo-ku, Chiba 260-8670, Japan

**Keywords:** residual cell, chemoresistance, metformin, lung cancer, gefitinib

## Abstract

Cancer chemotherapy, including molecular targeted therapy, has major limitations because it does not kill all the cancer cells; the residual cells survive until they acquire chemoresistance. In the present study, the combined effects of metformin and gefitinib were examined *in vivo* in a mouse xenograft model, inoculated with a human lung adenocarcinoma cell line that possesses an activating epidermal growth factor receptor mutation. The mechanism of the interaction was further elucidated *in vitro*. Metformin did not suppress the growth of already established tumors, nor did metformin augment tumor shrinkage by gefitinib. However, metformin significantly suppressed the regrowth of the tumor after effective treatment with gefitinib, suggesting the specific effect of metformin on the residual cells. Cytotoxicity of metformin was characterized by the absence of apoptosis induction and unremarkable cell cycle shift *in vitro*. The residual cell population after treatment with gefitinib was characterized by enriched cells with high expression of CD133 and CD24. Metformin was still effective on this specific cell population. Targeting residual cells after chemotherapy may represent an effective novel strategy for the treatment of cancer. Elucidating the mechanism of metformin cytotoxicity provides insights into future development of anticancer therapeutics.

## Introduction

Cytotoxic chemotherapy of most solid cancers rarely cures the cancer. Molecular targeted therapy has the same limitation, despite significantly enhanced cancer control and resulting prolonged survival, especially when cancers with driver mutations are treated with specific inhibitors, for example, when non-small cell lung cancer with an epidermal growth factor-receptor (*EGFR*) mutation is treated by EGFR-tyrosine kinase inhibitors (TKIs) (1). Thus, even after a complete response after a long period of treatment with gefitinib, the tumors grew again after withdrawal of the agent in an animal study (2). The secondary point mutation of T790M in *EGFR* (3) and establishment of a bypass signal transduction via *cMET* amplification (4) are known genetic alterations responsible for acquired EGFR-TKI resistance. If chemotherapy were sufficiently effective to kill the entire cell population of the tumor in a short period, however, the cancer would be cured before genetic adaptation and chemoresistance. Nevertheless, some cells in the tumor escape from effective chemotherapy and these residual cells survive until a chemoresistant phenotype is obtained. Therefore, complete elimination of residual cells would represent significant progress in cancer therapy. A recent study showed that the drug-tolerant phenotype, induced by acute response to chemotherapeutic agents, is reversible and that the phenotype maintains viability via engagement of insulin-like growth factor (IGF)-1 receptor signaling and an altered chromatin state that requires histone demethylase (5). This observation importantly provides a vision for a new strategy to treat cancer by specifically targeting the residual cells after chemotherapy.

Metformin is a safe biguanide that has been used worldwide to treat type 2 diabetes mellitus. Metformin activates AMP-activated protein kinase (AMPK), an enzyme that plays an important role in insulin signaling, whole body energy balance and the metabolism of glucose and fats, resulting in lowering of blood glucose (6). Metformin recently attracted attention for its potential anticancer effects (7). Epidemiological studies (8–10) first suggested a link between metformin and cancer prevention by demonstrating a lower incidence of death from cancer in patients with diabetes mellitus treated with metformin than those treated with other antidiabetic agents. These studies were followed by clinical observations, suggesting a link between metformin and increased pathologically complete response rate by induction chemotherapy in patients with breast cancer (10) as well as lower incidence rate of metastasis and a reduced risk of death in patients with lung cancer (11). These findings triggered a number of *in vitro* and *in vivo* experiments, revealing its antiproliferative properties in a variety of cancers (12–20). Although the precise mechanism is unclear, activation of AMPK might be crucial. First, liver kinase B1 (LKB1), a well-recognized tumor suppressor, activates AMPK (21,22) and metformin requires LKB1 for growth inhibitory action (23). Second, AMPK inhibits the mammalian target of rapamycin (mTOR) and the S6 kinase I pathways (24,25) and this inhibition appears to be achieved by phosphorylating tuberous sclerosis complex-2, another tumor suppressor and upstream regulator of mTOR (26). Notably, metformin blocks the growth-promoting effects of both insulin and IGF-1, deregulates AMPK activity and inhibits mTOR activity, S6 kinase activity and protein synthesis both in transformed and non-transformed mammary gland cells (14). However, it is unknown whether metformin causes apoptosis of cancer cells (13,17) or not (12,16), or whether metformin kills cancer cells synergistically with other cytotoxic agents (15,18,20,27) or antagonistically to cisplatin (28,29).

In the present study, *in vivo* experiments suggested a unique anticancer action for metformin, specifically on residual cells after chemotherapy. The mechanism was further elucidated with a series of *in vitro* experiments.

## Materials and methods

### Cell culture and reagents

A human lung adenocarcinoma cell line, PC9, purchased from Riken Cell Bank (accession no. RCB4455, Tsukuba, Japan), was used throughout the study. This cell line has an activating deletion of the *EGFR* gene (del E746–A750) in exon 19 (30). The cells were cultured as a monolayer in RPMI-1640 medium supplemented with 10% fetal bovine serum (FBS), 100 U/ml penicillin and 100 mg/ml streptomycin in a 37°C humidified atmosphere containing 5% CO_2_. Gefitinib (cat no. 3000, Tocris Bioscience, Ellisville, MO, USA) was dissolved in dimethyl sulfoxide (DMSO) and stored at −20°C until use. Metformin (1,1-dimethylbiguanide hydrochloride, cat no. D150959-5G, Sigma-Aldrich, St. Louis, MO, USA) was dissolved in phosphate buffered saline (PBS) at a concentration of 100 mM and stored at 4°C. A cisplatin solution at a concentration of 0.5 mg/ml (pH 2.5–5.5) was purchased from Nihon Kayaku (Tokyo, Japan). Each drug was diluted in the complete medium for each experiment and the final concentration of DMSO was <0.1%.

### Combined treatment of metformin and gefitinib in a mouse xenograft model

**Figure 1.**
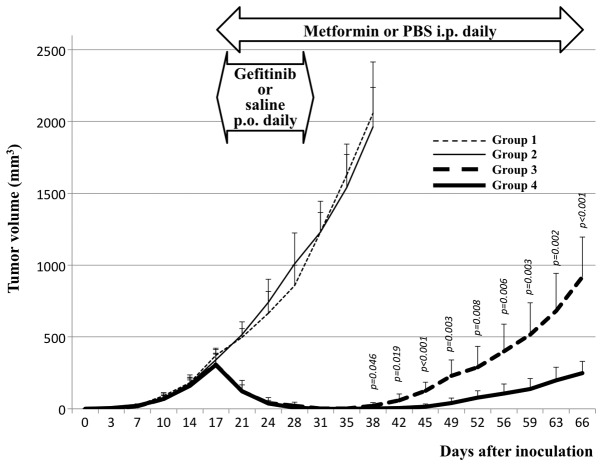
Effects of treatment with metformin, gefitinib and a combination of metformin and gefitinib, on the growth of PC9 xenograft tumors in SCID mice. After growing tumors for 16 days, the animals were randomly divided into 4 groups. In group 1 (fine broken line), saline (p.o.) was administered daily for 14 days (until day 30) and PBS (i.p.) was then administered daily until terminating the observation (day 66). In group 2 (fine, solid line), gefitinib (p.o., 150 mg/kg/day) suspended in saline was administered daily for 14 days and PBS (i.p.) was then administered daily until day 66. In group 3 (thick, broken line), saline (p.o.) was administered daily for 14 days and metformin (i.p., 250 mg/kg/day) dissolved in PBS was then administered daily until day 66. In group 4 (thick, solid line), both gefitinib and metformin were administered. The regrowth of the tumors after withdrawing gefitinib was significant, with each given p-value in the figure, suppressed by metformin (compare groups 3 and 4), whereas metformin exerted no effects on tumor growth (compare groups 1 and 2) and tumor shrinkage by gefitinib (compare groups 3 and 4). Each point represents the mean and the bars represent the SE (n=7).

Five to 6-week-old female severe combined immunodeficient (SCID) mice were acclimatized to local conditions for a week before starting the experiments. Aliquots of the cell suspension (2×10^6^ cells per mouse) were injected subcutaneously into their flanks. At day 16 (when the tumor volumes had reached ∼300 mm^3^), the mice were randomly allocated into 4 groups (7 mice per group). In every group, administration of either saline alone or gefitinib suspended in saline (150 mg/kg/day, every day, p.o. with gavage) and either PBS alone or metformin dissolved in PBS (250 mg/kg/day, every day, i.p.) were started. Either saline alone or gefitinib suspended in saline was continued for 14 days and either PBS alone or metformin dissolved in PBS was continued until terminating observation. In the first group, only saline (p.o.) and PBS (i.p.) were administered (control). In the second group, metformin dissolved in PBS was administered. In the third group, gefitinib suspended in saline was administered. In the fourth group, both gefitinib and metformin were administered (Fig. 1). The administration route of metformin was selected because a previous study showed that i.p. was better tolerated and more effective than p.o. *in vivo* (31). The dose of metformin was selected according to a preliminary experiment that showed that this dose was near maximal without causing death or body-weight loss in the animals (data not shown). The dose of gefitinib was previously published (32). In each animal, the tumor was allowed to grow until reaching ∼2,000 mm^3^, or until day 66, after which the animal was sacrificed. The tumor size was estimated by 2-dimensional caliper measurements and calculation with the formula π/6 × (A × B)^3/2^, where A and B represent the larger and smaller diameters of the tumor, respectively. The tumor size was measured twice a week during the observation period. The animal experiments were approved by the animal ethics review board of Chiba University (protocol no. A22-186) and were conducted in an animal facility at Chiba University under the strict SPF conditions in accordance with the established institutional guidelines.

### Cell proliferation in vitro

**Figure 2.**
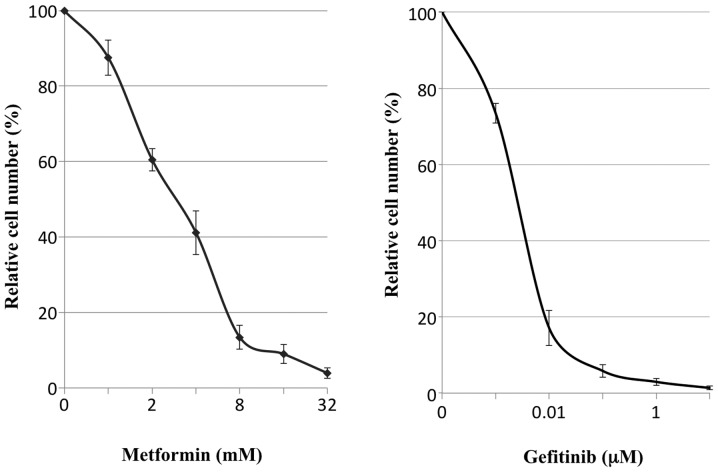
Dose-dependent growth inhibition of PC9 cells with metformin or gefitinib *in vitro*. The dots and bars represent the mean and SE (n=3), respectively.

For the *in vitro* chemosensitivity assay, 5×10^5^ cells per 6-cm-diameter culture dish were plated and cultured for 24 h until adding the agents metformin, gefitinib, cisplatin, or their combinations, to the medium for further culture. The cells were harvested, counted and the survival at defined time points (24, 48 and 72 h after adding the agents) was calculated. A series of preliminary experiments indicated that metformin at 10 mM, gefitinib at 0.03 *μ*M and cisplatin at 1.5 *μ*g/ml were nearly equivalent in reducing cell numbers to 10% of the cell number obtained without adding the agents (control) at 72 h (Fig. 2). Therefore, these concentrations were used for further experiments.

### Apoptosis

Apoptosis was evaluated by Hoechst staining and caspase activity determination. For Hoechst staining, cells treated with agents for 48 h were trypsinized and harvested together with the floating cells, fixed with 70% ethanol, stained with 2 *μ*g/ml bisbenzimide H33342 trihydrochloride (cat no. B2261-25MG; Sigma-Aldrich) and examined by fluorescence microscopy according to the manufacturer's instructions. Cells with aggregated or fragmented chromatin were regarded as apoptotic cells and 500 cells in each experiment were counted to calculate the apoptotic cell ratio. To determine the caspase activity in the cell extracts, a colorimetric assay was used to monitor the absorbance at 405 nm of *p*-nitroanilide (*p*NA) released from the synthetic substrates. Caspase 3 and 8 activities were evaluated with synthetic substrates DEVD-pNA and IETD-pNA, respectively, using the colorimetric assay kit APOPCYTO (Medical & Biological Laboratories, Nagoya, Japan) according to the manufacturer's instructions. In each assay, ∼200 *μ*g of protein was extracted from the cells treated with each agent for 24 h.

### Cell cycle

Cell cycle distributions were determined by a propidium iodide single-color flow cytometric method (FACSCanto II; BD Biosciences, San Jose, CA, USA), according to the manufacturer's instructions. Briefly, the cells were trypsinized, washed twice with ice-cold PBS, fixed with 70% ethanol and then stored at −20°C until analysis. Before analysis by FACS and CellQuest software (BD Biosciences), the cell suspensions were washed twice with PBS, suspended in 500 *μ*l of PI/RNase staining buffer (BD Biosciences) and incubated for 15 min at room temperature.

### Immunofluorescent staining for CD133

The cells were cultured in a chamber slide for 24 h, followed by treatment with the agents for 24 h. After removal of the medium containing the agents, the cells were fixed in a 1:1 mixture of methanol and acetone for 2 min, followed by blocking with normal goat serum for 30 min. The cells were incubated with primary anti-human CD133 antibodies (cat no. 130-090-422, Miltenyi Biotec, Bergisch Gladbach, Germany) overnight at 4°C. The cells were then washed 3 times in PBS and incubated with the secondary antibody (anti-mouse IgG) conjugated to the Alexa488 fluorescent dye for 1 h at room temperature. The stained cells were embedded in VectaShield mounting medium with DAPI (Vector Laboratories, Burlingame, CA, USA) and were examined with a Nikon Eclipse 80i microscope (Nikon, Tokyo, Japan) using the VB-7210 imaging system (Keyence, Tokyo, Japan). Staining results were directly observed at a magnification of ×200 using a fluorescence microscope.

### CD24 and CD44 expression determined by FACS

For analysis of cell-surface marker expression by FACS, anti-human CD24 antibodies conjugated to phycoerythrin (cat no. 311105, BioLegend, San Diego, CA, USA) and anti-human CD44 antibodies conjugated with allophycocyanin (cat no. 103011, BioLegend) were used. The cells treated with the agents for 24 h were trypsinized and washed 3 times with PBS. The cells (1×10^6^) in a single-cell suspension were resuspended in the staining buffer (PBS containing 2% FBS) and labeled with the antibodies, followed by washing and resuspension in 500 *μ*l of staining buffer according to the manufacturer's instructions. The cells were also labeled with propidium iodide to enrich viable cells and analyzed with a JSAN cell sorter and AppSan software (Bay Bioscience, Kobe, Japan).

### Enrichment of CD24-positive cells

To obtain a cell population enriched in CD24-positive cells, a magnetic cell-sorting system with the Miltenyi Biotec MACS Cell Separation kit was used according to the manufacturer's instructions. The sorted cells were analyzed with a JSAN cell sorter and the AppSan software as described above. The cell sorting and subsequent brief culture for propagation were repeated up to 4 times to obtain a cell population consisting of ∼80% CD24-positive cells.

### Statistical analysis

Data are presented as the means ± SEs as indicated and were analyzed by Student's t-test. A p-value of <0.05 (2-tailed) was considered statistically significant.

## Results

### Effect of gefitinib and metformin on xenograft tumors in vivo

When the administration was initiated day 16 or when the tumor size reached ∼300 mm^3^, metformin did not reduce tumor growth (compare groups 2 to 1, Fig. 1). In addition, no additional effect was observed with metformin in tumor shrinkage with gefitinib (see the curves from days 16 to 31 in groups 3 and 4). Metformin, however, significantly reduced tumor regrowth after withdrawal of gefitinib treatment (see the curves from days 35 to 66 in groups 3 and 4).

### Suppression of in vitro cell proliferation

**Figure 3.**
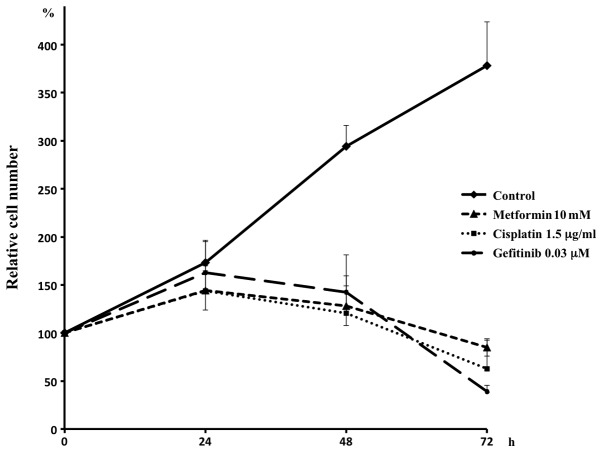
Growth curves of PC9 cells *in vitro*. In contrast to the cells that grew to 380% of the originally plated cell number without any agent after 72 h of culture, metformin, gefitinib and cisplatin suppressed cell growth. Because the concentrations of the agents adopted here were almost equivalent in suppression, they were used for further experiments. The dots and bars represent the mean and SE (n=3), respectively.

*In vitro* administration of metformin suppressed proliferation of PC9 cells in a dose-dependent manner, similarly to gefitinib (Fig. 2) and the concentrations that resulted in 90% reduction of cell numbers compared with controls at 72 h after the administration were 11.6±1.87 mM with metformin, 0.042±0.024 *μ*M with gefitinib and 1.48±0.18 *μ*g/ml with cisplatin. In subsequent experiments, concentrations of 10 mM for metformin, 0.03 *μ*M for gefitinib and 1.5 *μ*g/ml for cisplatin were employed (Fig. 3).

### Apoptosis induction by the agents

**Figure 4.**
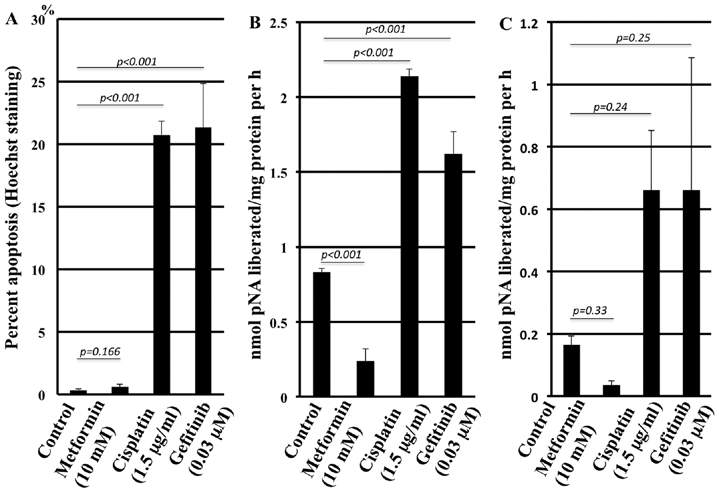
Apoptotic cells were counted by Hoechst staining 48 h after exposure to the agents. Percentage of apoptotic cells per total cells was determined (A). Apoptosis was also assessed by caspase 3 (B) and caspase 8 (C) activities, 24 h after exposure to the agents. The columns and bars represent the mean and SE (n=3), respectively.

**Figure 5.**
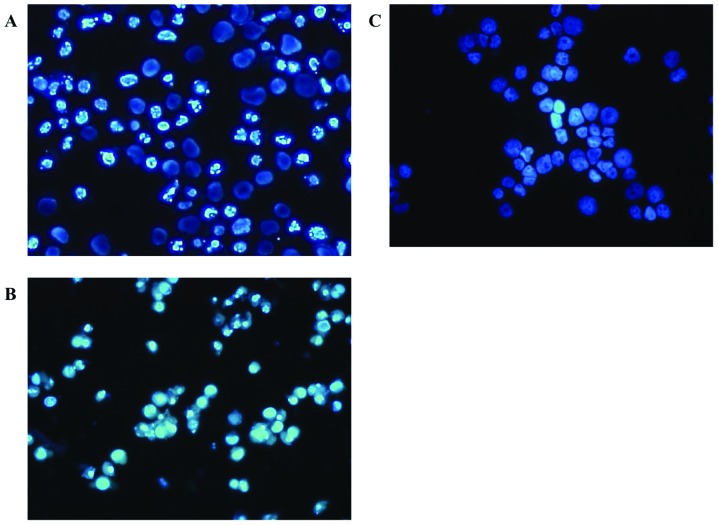
Apoptotic cells were counted by Hoechst staining 48 h after exposure to the agents. Representative morphology in the presence of cisplatin (A), gefitinib (B) and metformin (C) is shown. Metformin failed to induce apoptosis in contrast to cisplatin and gefitinib.

As assessed by Hoechst staining, metformin did not induce more apoptotic cells compared with the control in contrast to cisplatin and gefitinib (Fig. 4A). The representative morphology in the presence of the agents is shown in Fig. 5. The caspase 3 assay confirmed the results (Fig. 4B) with statistically significant differences, although the caspase 8 assay solely revealed a trend without statistical significance (Fig. 4C).

### Cell cycle alteration

**Figure 6.**
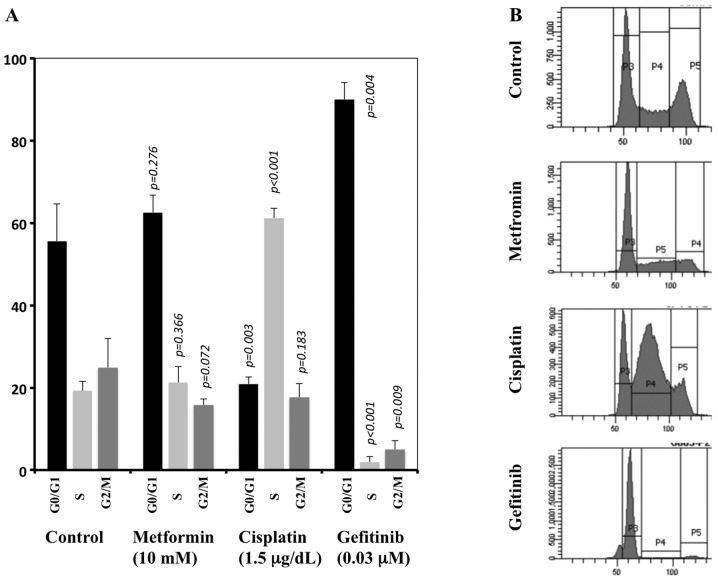
Altered cell cycle distributions 24 h after exposure to the agents, assessed by FACS. The cell cycle distribution was not significantly altered with metformin, in contrast with cisplatin and gefitinib. The columns and bars represent the mean and SE [n=3, (A)], respectively. The p-value for difference between each column and the corresponding column in the control is given. A representative FACS analysis with each agent is shown (B).

FACS analysis showed that metformin did not significantly alter the cell cycle distribution compared to the control. In contrast, cisplatin caused a marked cell accumulation at the S phase together with a marked decrease of cells at the G0/G1 phases. Gefitinib caused marked accumulation at the G0/G1 phases together with a marked decrease at the S and G2/M phases compared with the control (Fig. 6).

### Expression of CD24, CD44 and CD133 in surviving cells after exposure to gefitinib

**Figure 7.**
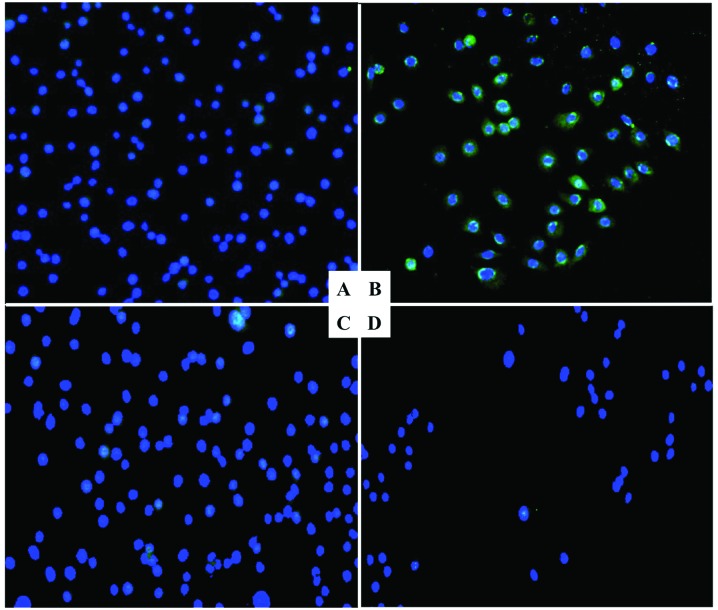
Immunofluorescent staining of CD133 in cells. Compared with the untreated cells (A), the population treated by gefitinib was enriched with CD133 positive cells (B). In contrast, metformin treatment did not enrich CD133 positive cells (C). When the cells were treated with both agents, no enrichment was observed (D).

**Figure 8.**
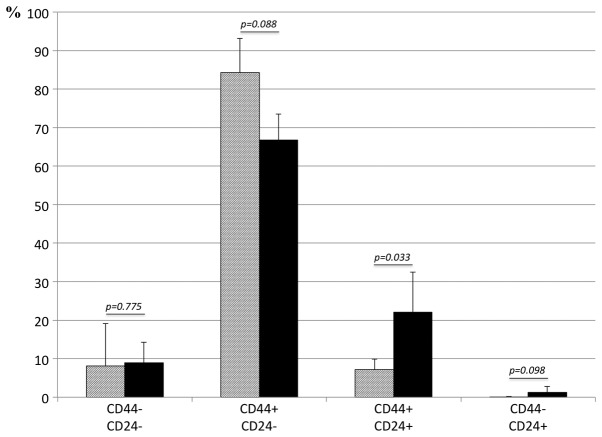
FACS analysis for altered expression of CD24 and CD44 after gefitinib treatment. The shaded and closed columns represent the mean percentages of the untreated and treated cells, respectively. The bars represent the SE (n=3). CD24 expressing cells significantly increased during gefitinib treatment, from ∼8–23%, with CD44 expressing cells unaltered.

Because we failed to detect reproducible CD133 expression by FACS analysis, CD133 expression was assessed solely by immunofluorescence staining. The CD133-positive cells were significantly enriched after exposure to gefitinib for 24 h. In contrast, exposure to metformin alone or to gefitinib combined with metformin did not augment CD133 expression (Fig. 7). Altered expression of CD24 and CD44, assessed by FACS, are shown in Fig. 8. The cell population with negative staining for CD44, either with or without CD24 expression, was not significantly altered by exposure to gefitinib for 24 h. The cell population with positive staining for CD24 increased significantly after exposure to gefitinib for 24 h.

### Sensitivity to metformin and gefitinib in CD24-positive cells

**Figure 9.**
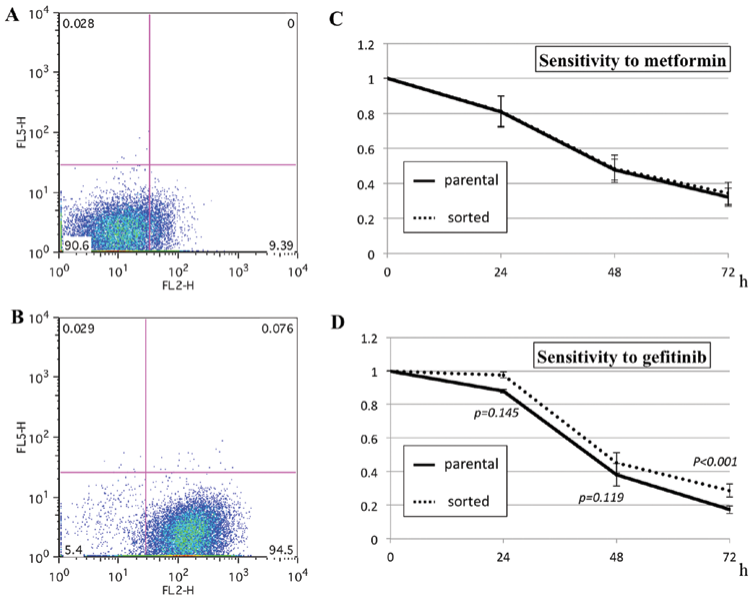
The proportion of CD24-positive cells [the right lower squares in (A and B)] increased from 7.1±4.2% (n=3) in the parental cells (A) to 81.8±12.1% (n=3) in the sorted cells (B). The sensitivity to metformin (C) and gefitinib (D) of these cells were compared *in vitro*.

To obtain a cell population enriched in CD24-positive cells, the parental cells were sorted by FACS magnetic separation. Because a single sorting was not sufficient to enrich CD24-positive cells, the sorting was repeated up to 4 times. The proportion of CD24-positive cells in the parental cells (7.1±4.2%, n=3) was enriched to 81.8±12.1% (n=3). The *in vitro* sensitivity assay revealed that the parental and sorted cells showed nearly identical sensitivity to metformin. The sorted cells, however, were slightly but significantly more resistant to gefitinib than the parental cells (Fig. 9).

## Discussion

The present study employed the human lung adenocarcinoma cell line PC9, which possesses an *EGFR* exon 19 deletion mutation that renders EGFR sensitive to the TKIs. *In vivo* experiments with xenografts derived from these cells resulted in the following observations: i) metformin exerted no effect on already grown tumors (>300 mm^3^), ii) metformin had no additional effect on tumor shrinkage by gefitinib, iii) the tumors regrew after withdrawing gefitinib even after the treatment had resulted in complete regression of the tumors and iv) metformin significantly suppressed the regrowth of the tumors after withdrawing gefitinib treatment. These observations suggest that metformin is effective specifically on residual cells after gefitinib treatment; however, metformin is not sufficiently effective to suppress growth of already established tumors.

To test our hypothesis, a series of *in vitro* experiments were conducted. Cisplatin was included as a positive control in some of the experiments. A dose that resulted in an *in vitro* cell number reduction to 10% of the original cell number was chosen for each agent. Metformin did not induce apoptosis when assessed by Hoechst staining and caspase 3 and 8 activity determination, in contrast to gefitinib and cisplatin. Moreover, apoptosis induction by metformin treatment was significantly lower, even lower than what was observed in the control experiments, suggesting an apoptosis-protective property of metformin. This is consistent with a previous report that demonstrated a preventive effect of AMPK on apoptosis (33,34). Although metformin decreased the cells at the G2/M phases, cell cycle alteration with metformin was not dramatic in contrast with cisplatin and gefitinib, which induced significant accumulation at the S and G0/G1 phases, respectively. Although the results obtained with cisplatin (35) and gefitinib (36,37) are consistent with previously reported data, the results obtained with metformin are rather complicated. Some studies reported an absence of apoptosis induction with metformin (17), whereas other reports described a significant apoptosis induction (12). Similarly, although some have reported a significant cell cycle shift with metformin (12,16), others have reported only mild cell cycle arrest at the G0-G1 phases in the presence of metformin (38,39). The effects of metformin on apoptosis and cell cycle arrest may vary depending on the cell line examined, as previously reported with human lung cancers of a variety of histological types (40). Nevertheless, the minimum effects of metformin on apoptosis induction and cell cycle alteration *in vitro* in the present experimental system may explain the absence of effects on tumor growth inhibition *in vivo*.

Based on the reported drug resistance of cancer stem cells (41) and the information presented in a previous study (5), the expression of 3 putative cancer stem cell markers, CD133 (42), CD44 (43) and CD24 (44), was examined. After gefitinib treatment of the cells *in vitro*, cells with CD133 expression were enriched as assessed by immunofluorescence staining. FACS analysis revealed an enrichment of cells with CD24 expression after gefitinib treatment *in vitro*. The population of cells with CD44 expression was unaltered. These observations are similar to those in a previous report (5), suggesting that cells with CD133 or CD24 expression may be resistant to gefitinib. In fact, metformin treatment *in vitro* did not enrich cells with CD133 expression, in contrast to what was observed with gefitinib. In addition, combined treatment with metformin and gefitinib canceled the enrichment observed in the treatment with gefitinib alone. These results strongly suggest that metformin is effective against residual cells after *in vitro* treatment with gefitinib, consistent with the *in vivo* experiment. A cell population consisting of ∼80% cells expressing CD24 (∼10-fold enrichment compared with the parental cells) was obtained by FACS sorting to directly examine chemosensitivity. These cells were slightly but significantly more resistant to gefitinib than the parental cells, whereas their sensitivity to metformin was identical to the parental cells, suggesting that metformin was effective against residual cells after gefitinib treatment. Nevertheless, the degree of augmented resistance to gefitinib in cells with CD24 expression was unexpectedly small. This can be explained if the resistant cells express CD24 and if only a part of the cell population with CD24 expression is resistant to gefitinib.

The nature and properties of the residual cells after treatment with gefitinib are unclear. Although CD133 and CD24 are putative markers for cancer stem cells in human brain and colon cancers, respectively, the present results do not indicate that the residual cells are cancer stem cells because cancer stem cells in human lung cancer have not yet been identified. Considering their prompt emergence in a short period, non-mutational mechanisms, including epigenetic change and selection of resistant cells from a heterogeneous cell population, seem to be the most likely routes of chemo-resistance. Cancer stem cells and epithelial-mesenchymal transitions might be possible candidates for selection. Specific targeting of residual cells after chemotherapy may be a suitable approach to cure cancers. The present study highlighted metformin as a candidate for targeting residual cells and we envision that further elucidation of the detailed molecular mechanism of the cytotoxicity of metformin represents progress in cancer therapy.

## References

[1] Inoue A, Kobayashi K, Maemondo M (2012). Updated overall survival results from a randomized phase III trial comparing gefitinib with carboplatin-paclitaxel for chemo-naive non-small cell lung cancer with sensitive EGFR gene mutations (NEJ002). Ann Oncol.

[2] Wakeling AE, Guy SP, Woodburn JR (2002). ZD1839 (Iressa): an orally active inhibitor of epidermal growth factor signaling with potential for cancer therapy. Cancer Res.

[3] Kosaka T, Yatabe Y, Endoh H (2006). Analysis of epidermal growth factor receptor gene mutation in patients with non-small cell lung cancer and acquired resistance to gefitinib. Clin Cancer Res.

[4] Engelman JA, Zejnullahu K, Mitsudomi T (2007). MET amplification leads to gefitinib resistance in lung cancer by activating ERBB3 signaling. Science.

[5] Sharma SV, Lee DY, Li B (2010). A chromatin-mediated reversible drug-tolerant state in cancer cell subpopulations. Cell.

[6] Towler MC, Hardie DG (2007). AMP-activated protein kinase in metabolic control and insulin signaling. Circ Res.

[7] Pollak MN (2012). Investigating metformin for cancer prevention and treatment: the end of the beginning. Cancer Discov.

[8] Evans JM, Donnelly LA, Emslie-Smith AM, Alessi DR, Morris AD (2005). Metformin and reduced risk of cancer in diabetic patients. BMJ.

[9] Bowker SL, Majumdar SR, Veugelers P, Johnson JA (2006). Increased cancer-related mortality for patients with type 2 diabetes who use sulfonylureas or insulin. Diabetes Care.

[10] Jiralerspong S, Palla SL, Giordano SH (2009). Metformin and pathologic complete responses to neoadjuvant chemotherapy in diabetic patients with breast cancer. J Clin Oncol.

[11] Mazzone PJ, Rai H, Beukemann M, Xu M, Jain A, Sasidhar M (2012). The effect of metformin and thiazolidinedione use on lung cancer in diabetics. BMC Cancer.

[12] Alimova IN, Liu B, Fan Z (2009). Metformin inhibits breast cancer cell growth, colony formation and induces cell cycle arrest in vitro. Cell Cycle.

[13] Liu B, Fan Z, Edgerton SM (2009). Metformin induces unique biological and molecular responses in triple negative breast cancer cells. Cell Cycle.

[14] Zakikhani M, Dowling R, Fantus IG, Sonenberg N, Pollak M (2006). Metformin is an AMP kinase-dependent growth inhibitor for breast cancer cells. Cancer Res.

[15] Rocha GZ, Dias MM, Ropelle ER (2011). Metformin amplifies chemotherapy-induced AMPK activation and antitumoral growth. Clin Cancer Res.

[16] Ben Sahra I, Laurent K, Loubat A (2008). The antidiabetic drug metformin exerts an antitumoral effect in vitro and in vivo through a decrease of cyclin D1 level. Oncogene.

[17] Wang LW, Li ZS, Zou DW, Jin ZD, Gao J, Xu GM (2008). Metformin induces apoptosis of pancreatic cancer cells. World J Gastroenterol.

[18] Gotlieb WH, Saumet J, Beauchamp MC (2008). In vitro metformin anti-neoplastic activity in epithelial ovarian cancer. Gynecol Oncol.

[19] Rattan R, Giri S, Hartmann LC, Shridhar V (2011). Metformin attenuates ovarian cancer cell growth in an AMP-kinase dispensable manner. J Cell Mol Med.

[20] Rattan R, Graham RP, Maguire JL, Giri S, Shridhar V (2011). Metformin suppresses ovarian cancer growth and metastasis with enhancement of cisplatin cytotoxicity in vivo. Neoplasia.

[21] Hawley SA, Boudeau J, Reid JL (2003). Complexes between the LKB1 tumor suppressor, STRAD alpha/beta and MO25 alpha/beta are upstream kinases in the AMP-activated protein kinase cascade. J Biol.

[22] Lizcano JM, Goransson O, Toth R (2004). LKB1 is a master kinase that activates 13 kinases of the AMPK subfamily, including MARK/PAR-1. EMBO J.

[23] Dowling RJ, Zakikhani M, Fantus IG, Pollak M, Sonenberg N (2007). Metformin inhibits mammalian target of rapamycin-dependent translation initiation in breast cancer cells. Cancer Res.

[24] Bolster DR, Crozier SJ, Kimball SR, Jefferson LS (2002). AMP-activated protein kinase suppresses protein synthesis in rat skeletal muscle through down-regulated mammalian target of rapamycin (mTOR) signaling. J Biol Chem.

[25] Kimura N, Tokunaga C, Dalal S (2003). A possible linkage between AMP-activated protein kinase (AMPK) and mammalian target of rapamycin (mTOR) signalling pathway. Genes Cells.

[26] Inoki K, Zhu T, Guan KL (2003). TSC2 mediates cellular energy response to control cell growth and survival. Cell.

[27] Iliopoulos D, Hirsch HA, Struhl K (2011). Metformin decreases the dose of chemotherapy for prolonging tumor remission in mouse xenografts involving multiple cancer cell types. Cancer Res.

[28] Janjetovic K, Vucicevic L, Misirkic M (2011). Metformin reduces cisplatin-mediated apoptotic death of cancer cells through AMPK-independent activation of Akt. Eur J Pharmacol.

[29] Harhaji-Trajkovic L, Vilimanovich U, Kravic-Stevovic T, Bumbasirevic V, Trajkovic V (2009). AMPK-mediated autophagy inhibits apoptosis in cisplatin-treated tumour cells. J Cell Mol Med.

[30] Costa DB, Halmos B, Kumar A (2007). BIM mediates EGFR tyrosine kinase inhibitor-induced apoptosis in lung cancers with oncogenic EGFR mutations. PLoS Med.

[31] Memmott RM, Mercado JR, Maier CR, Kawabata S, Fox SD, Dennis PA (2010). Metformin prevents tobacco carcinogen-induced lung tumorigenesis. Cancer Prev Res.

[32] Sirotnak FM, Zakowski MF, Miller VA, Scher HI, Kris MG (2000). Efficacy of cytotoxic agents against human tumor xenografts is markedly enhanced by coadministration of ZD1839 (Iressa), an inhibitor of EGFR tyrosine kinase. Clin Cancer Res.

[33] Blazquez C, Geelen MJ, Velasco G, Guzman M (2001). The AMP-activated protein kinase prevents ceramide synthesis de novo and apoptosis in astrocytes. FEBS Lett.

[34] Shaw RJ, Kosmatka M, Bardeesy N (2004). The tumor suppressor LKB1 kinase directly activates AMP-activated kinase and regulates apoptosis in response to energy stress. Proc Natl Acad Sci USA.

[35] Ormerod MG, Orr RM, Peacock JH (1994). The role of apoptosis in cell killing by cisplatin: a flow cytometric study. Br J Cancer.

[36] Janmaat ML, Kruyt FA, Rodriguez JA, Giaccone G (2003). Response to epidermal growth factor receptor inhibitors in non-small cell lung cancer cells: limited antiproliferative effects and absence of apoptosis associated with persistent activity of extracellular signal-regulated kinase or Akt kinase pathways. Clin Cancer Res.

[37] Tracy S, Mukohara T, Hansen M, Meyerson M, Johnson BE, Janne PA (2004). Gefitinib induces apoptosis in the EGFRL858R non-small-cell lung cancer cell line H3255. Cancer Res.

[38] Cantrell LA, Zhou C, Mendivil A, Malloy KM, Gehrig PA, Bae-Jump VL (2010). Metformin is a potent inhibitor of endometrial cancer cell proliferation - implications for a novel treatment strategy. Gynecol Oncol.

[39] Kato K, Gong J, Iwama H (2012). The antidiabetic drug metformin inhibits gastric cancer cell proliferation in vitro and in vivo. Mol Cancer Ther.

[40] Ashinuma H, Takiguchi Y, Kitazono S (2012). Antiproliferative action of metformin in human lung cancer cell lines. Oncol Rep.

[41] Matsui W, Wang Q, Barber JP (2008). Clonogenic multiple myeloma progenitors, stem cell properties, and drug resistance. Cancer Res.

[42] Hemmati HD, Nakano I, Lazareff JA (2003). Cancerous stem cells can arise from pediatric brain tumors. Proc Natl Acad Sci USA.

[43] Al-Hajj M, Wicha MS, Benito-Hernandez A, Morrison SJ, Clarke MF (2003). Prospective identification of tumorigenic breast cancer cells. Proc Natl Acad Sci USA.

[44] Vermeulen L, Todaro M, de Sousa Mello F (2008). Single-cell cloning of colon cancer stem cells reveals a multi-lineage differentiation capacity. Proc Natl Acad Sci USA.

